# Modeling the potential health impact of prospective Strep A vaccines

**DOI:** 10.1038/s41541-023-00668-0

**Published:** 2023-06-10

**Authors:** Fiona Giannini, Jeffrey W. Cannon, Daniel Cadarette, David E. Bloom, Hannah C. Moore, Jonathan Carapetis, Kaja Abbas

**Affiliations:** 1 Wesfarmers Centre for Vaccines and Infectious Diseases, Telethon Kids Institute, University of Western Australia, Perth, Australia; 2 Harvard T.H. Chan School of Public Health, Boston, USA; 3 School of Population Health, Curtin University, Perth, Australia; 4 Perth Children’s Hospital, Perth, Australia; 5 London School of Hygiene & Tropical Medicine, London, UK

**Keywords:** Diseases, Epidemiology, Epidemiology

## Abstract

The World Health Organization published the preferred product characteristics for a Group A *Streptococcus* (Strep A) vaccine in 2018. Based on these parameters for the age of vaccination, vaccine efficacy, duration of protection from vaccine-derived immunity, and vaccination coverage, we developed a static cohort model to estimate the projected health impact of Strep A vaccination at the global, regional, and national levels and by country-income category. We used the model to analyse six strategic scenarios. Based on Strep A vaccine introduction between 2022 and 2034 for the primary scenario, we estimated vaccination at birth for 30 vaccinated cohorts could avert 2.5 billion episodes of pharyngitis, 354 million episodes of impetigo, 1.4 million episodes of invasive disease, 24 million episodes of cellulitis, and 6 million cases of rheumatic heart disease globally. Vaccination impact in terms of burden averted per fully vaccinated individual is highest in North America for cellulitis and in Sub-Saharan Africa for rheumatic heart disease.

## Introduction

Group A *Streptococcus* (Strep A) infection and direct sequelae are a major cause of morbidity and mortality at the global level, with more than half a million deaths annually and 1.78 million new cases each year attributable to Strep A^1–4^. Strep A causes a broad spectrum of diseases, including pharyngitis, impetigo (skin infections), invasive disease, cellulitis, rheumatic heart disease (RHD), acute rheumatic fever (ARF), and acute post-streptococcal glomerulonephritis (APSGN / kidney disease). Children and young adults, particularly those in low- and middle-income countries, are the most affected by ARF and RHD^5,6^, which impose substantial health burden in the form of morbidity and premature mortality due to cardiovascular impairment, as well as a substantial economic burden at the individual, household, and societal levels^7^. Invasive Strep A infections peak in infants and adults 65 years and older, with high case-fatality rates in the older age group^8^. The rate of cellulitis, a frequent precursor to invasive infection, increases with age and a higher proportion of cases require hospitalisation^9,10^. While relatively mild compared with RHD and invasive infection, cases and suspected cases of Strep A pharyngitis are most frequent in school-aged children and are major drivers of antibiotic use and misuse^11,12^, which can contribute to antimicrobial resistance in both Strep A and off-target (bystander) pathogens. While Strep A transmission occurs primarily through infections in the mucosae and skin, the relative impact of individual transmission modes, such as via large respiratory droplets, skin-to-skin contact, and fomites, has not been studied holistically^13^.

Select diseases are identified as WHO (World Health Organization) priorities due to their unmet public health need for vaccines, assessment of technical feasibility, and suitability for use in low- and middle-income countries^14^. The global mortality and morbidity burden associated with Strep A infection and direct sequelae are substantially high to support the public health need for the development of new Strep A vaccines. The WHO published the Preferred Product Characteristics (PPC) for Strep A vaccines in 2018^15^. The WHO Research and Development Technology Roadmap and the PPC describe the preferred parameters related to Strep A vaccine indications, target population, data needs for safety and efficacy evaluation, research and development, and vaccination strategies^14,15^. The WHO vision for Strep A vaccines and the near-term strategic goal is to demonstrate good safety and proof of efficacy of a candidate Strep A vaccine against pharyngitis and impetigo in children. The long-term strategic goal is to develop a safe, globally effective, and affordable Strep A vaccine to prevent acute Strep A infections (pharyngitis, impetigo, invasive disease, and cellulitis), avert associated antibiotic use and prevent secondary immune-mediated sequelae (APSGN, ARF, and RHD) and associated mortality.

Based on the WHO’s preferred product characteristics, we developed a mathematical model to assess the health impact of vaccination at the global, regional, and national levels and by country-income category. We used this Strep A vaccine impact model to analyse strategic scenarios for varied ages of vaccination (at birth or 5 years of age), vaccine efficacy, dynamics and duration of protection from vaccine-derived immunity, and vaccination coverage. To our knowledge, our model is the first to be calibrated to predict a range of Strep A diseases for >200 countries. Previous models have been limited in the scope of predicted diseases (e.g., models for RHD given preceding Strep A pharyngitis)^16^ or in geography, namely models for Australia and New Zealand^17,18^.

Box 1 Key findings

- Around one-third of the annual global burden of rheumatic heart disease could potentially be averted by Strep A vaccination.
- Vaccination impact by region is estimated to be highest in North America for cellulitis and in Sub-Saharan Africa for rheumatic heart disease.
- By income level, total vaccine-avertable Strep A disease burdens were highest in lower-middle income countries.

## Results

We developed the Strep A vaccine impact model using the R statistical software^19^ and included a user-friendly R Shiny web application. The program code and data for the vaccine impact model is available as an R package, *GASImpactModel* (https://github.com/fionagi/GASImpactModel) and modeling analysis can be conducted through the R Shiny web application (https://github.com/fionagi/GASImpactModel_App). Through the web application, the impact of a selected vaccination scenario can be visualized for any of 205 countries. The app shows the predicted lifetime health benefits from age of vaccination associated with the vaccination of multiple cohorts during the period selected. The vaccine impact model can estimate the health benefits of vaccination by calendar year, birth year, and year of vaccination^20^.

**Fig. 1:**
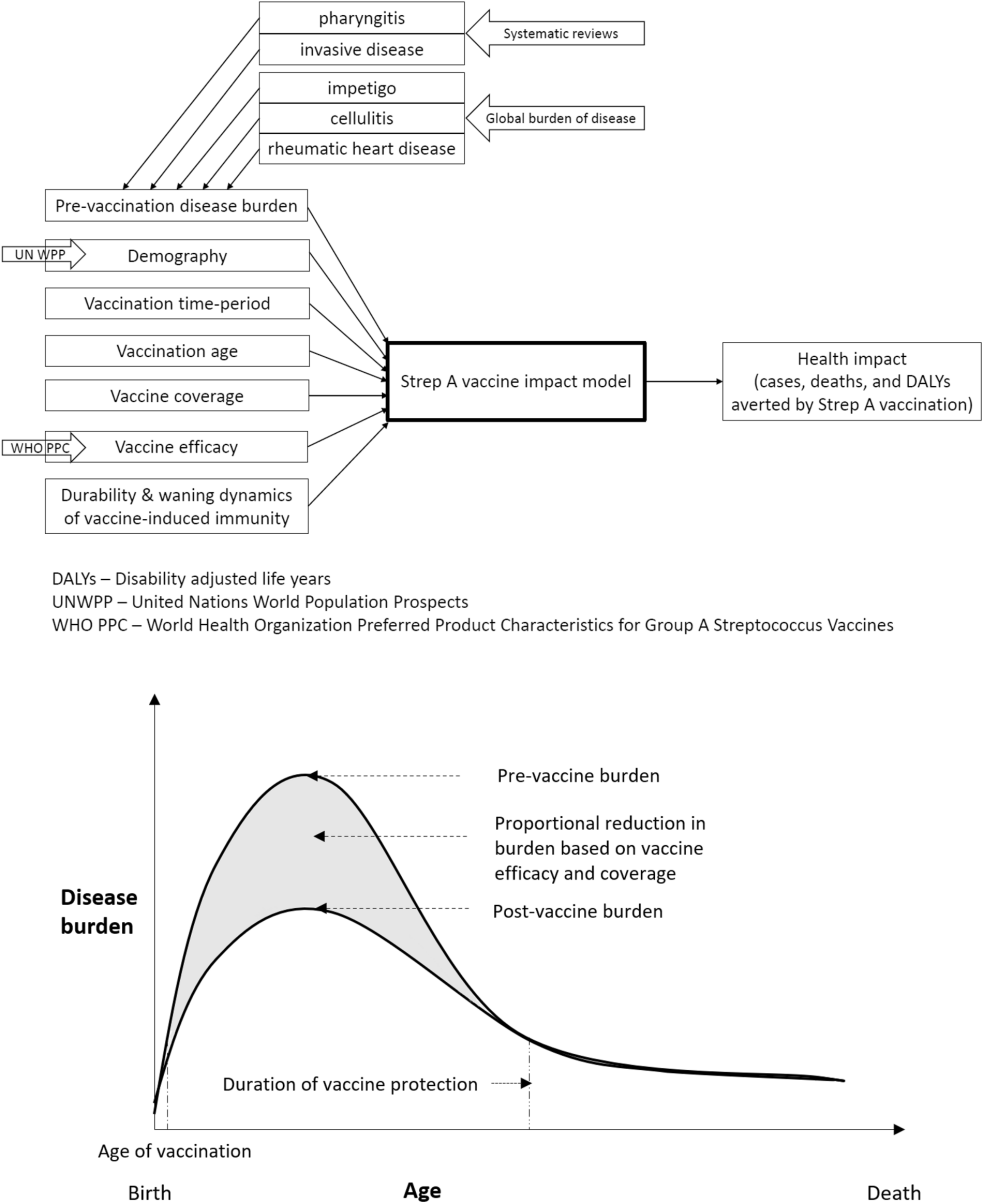
Strep A vaccine impact model. The static cohort model estimates the projected health impact of Strep A vaccination among the vaccinated cohorts over their lifetime in terms of burden averted for pharyngitis, invasive disease, impetigo, cellulitis, and rheumatic heart disease.

**Fig. 2:**
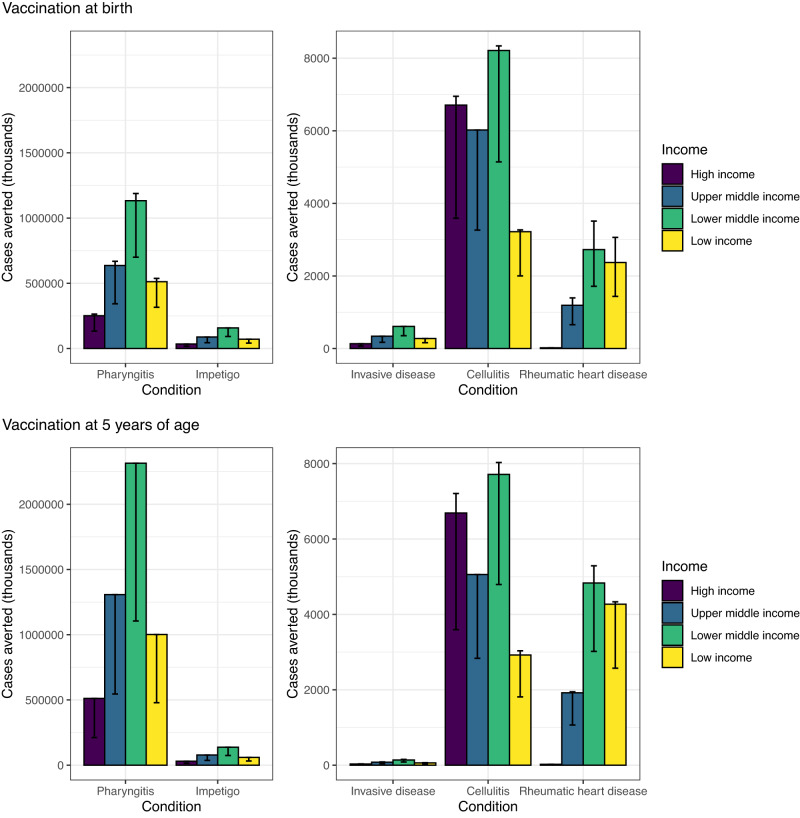
Vaccine impact at the country-income levels. The vaccine impact on cases averted (in thousands) is stratified by income levels of countries (World Bank income classification), based on the lifetime health impact of vaccination at birth or 5 years of age for 30 vaccinated cohorts from year of vaccine introduction on Strep A disease burden (pharyngitis, impetigo, invasive disease, cellulitis, and rheumatic heart disease). The vertical bars show the estimates for scenario 1, and the error bars show the range across scenarios 1-6. Note the difference in scale between the left panel (pharyngitis and impetigo) and the right panel (invasive, cellulitis and rheumatic heart disease).

**Fig. 3:**
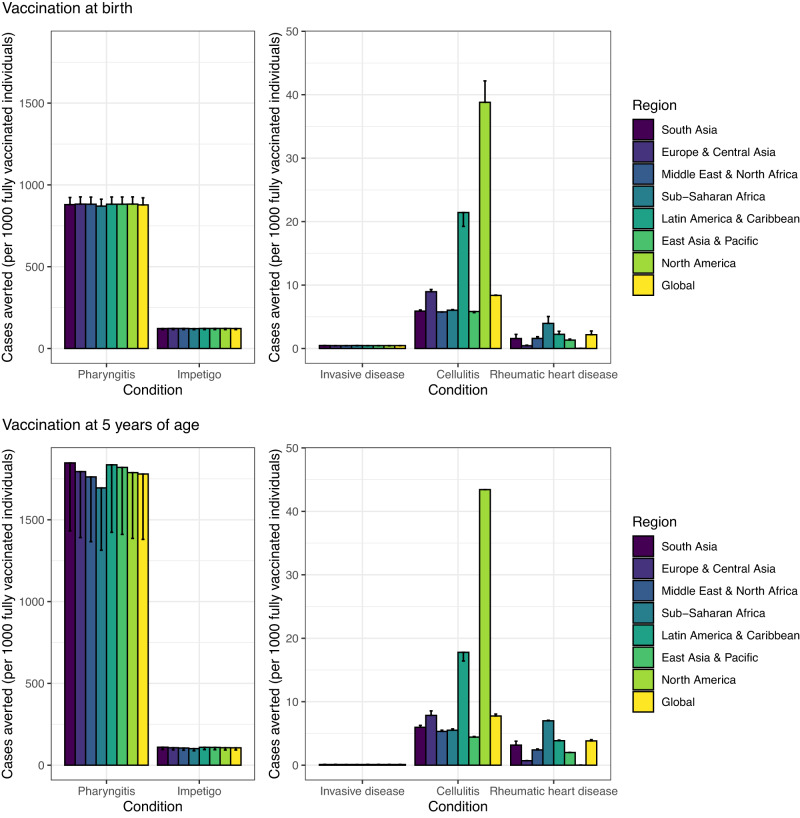
Vaccine impact at the regional and global levels. The vaccine impact on cases averted per 1000 fully vaccinated individuals is stratified at the regional (United Nations regions) and global levels for different scenarios (estimate for scenario 1 and range across the six scenarios), based on the lifetime health impact of vaccination at birth or 5 years of age for 30 vaccinated cohorts from year of vaccine introduction on Strep A disease burden (pharyngitis, impetigo, invasive disease, cellulitis, and rheumatic heart disease). The vertical bars show the estimates for scenarios 1, 3, and 5 (which are equal), and the error bars show the estimates for scenarios 2, 4, and 6 (which are equal). Note the difference in scale between the left panel (pharyngitis and impetigo) and the right panel (invasive, cellulitis and rheumatic heart disease).

**Fig. 4:**
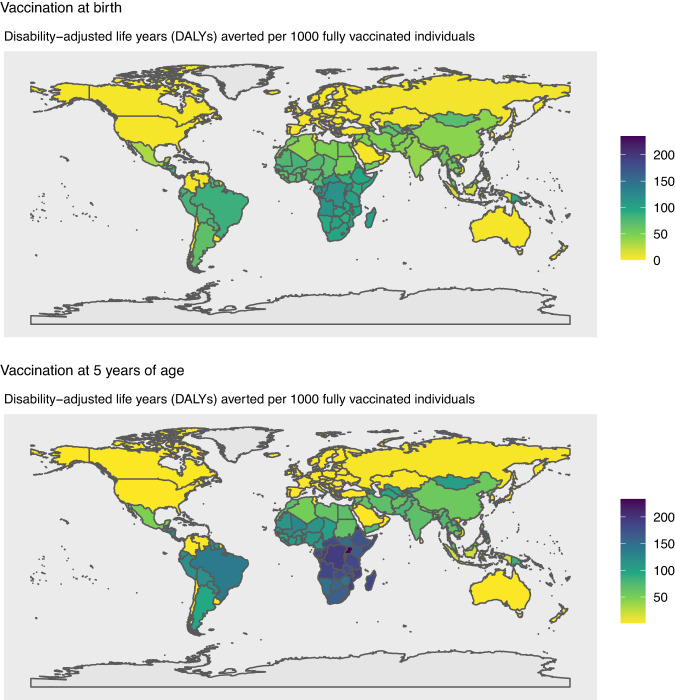
Vaccine impact at the national level. The vaccine impact on disability-adjusted life years (DALYs) averted per 1000 fully vaccinated individuals is shown for 183 countries, based on the lifetime health impact of vaccination at birth or 5 years of age for 30 vaccinated cohorts from year of vaccine introduction on Strep A disease burden (pharyngitis, impetigo, invasive disease, cellulitis, and rheumatic heart disease) for scenario 1.

We used the vaccine efficacy assumptions (see Table 1) from the WHO preferred product characteristics for a Strep A vaccine and analysed six strategic scenarios (see Table 2) for varied years of vaccine introduction, coverage, and waning dynamics using the Strep A vaccine impact model (see Fig. 1). For pre-vaccination, the baseline Strep A burden for pharyngitis and invasive disease are informed by systematic reviews^21,22^, and for impetigo, cellulitis, and rheumatic heart disease from the Global Burden of Disease studies^23^. Table 3 shows the baseline cases of Strep A disease burden before the introduction of vaccination. Tables 4 and 5 and Figs. 2–4 present the health impact of Strep A vaccination on lifetime disease burden averted among the vaccinated cohorts for the different scenarios. Box 1 highlights our key findings.

**Table 1:** Vaccine efficacy.

Strep A disease state/sequelae	Vaccine efficacy (%)
Pharyngitis	80
Impetigo	80
Invasive disease	70
Cellulitis	70
Rheumatic heart disease	50

The vaccine efficacy assumptions are based on the WHO preferred product characteristics for the Strep A vaccine.

**Table 2:** Vaccination scenarios.

Scenario	Year of vaccine introduction	Maximum coverage	Durability of vaccine-derived immunity
1	Country-specific (2022 - 2034)	Country-specific (9 - 99%)	Full efficacy for 10 years
2	Country-specific (2022 - 2034)	Country-specific (9 - 99%)	Linear waning over 20 years
3	2022	50%	Full efficacy for 10 years
4	2022	50%	Linear waning over 20 years
5	Country-specific (2022 - 2034)	50%	Full efficacy for 10 years
6	Country-specific (2022 - 2034)	50%	Linear waning over 20 years

Potential vaccination scenarios for varying years of vaccine introduction, coverage, vaccine-derived immunity dynamics, and age of vaccination (at birth or 5 years of age).

**Table 3:** Baseline cases at the regional and global levels.

UN regions	Scenarios	Cases (thousands)									
		Pharyngitis		Impetigo		Invasive disease		Cellulitis		Rheumatic heart disease	
**South Asia**	1,2,5,6	997,239	1,992,706	139,217	211,562	612	728	7642	14,792	2893	10,741
	3,4	1,014,494	2,027,050	141,670	215,255	623	741	7777	15,050	2939	10,907
**Europe & Central Asia**	1,2,5,6	316,919	633,687	44,127	67,156	193	230	3747	7211	249	654
	3,4	322,505	644,834	44,909	68,342	196	234	3805	7324	249	654
**Middle East & North Africa**	1,2,5,6	314,558	628,787	43,838	66,679	192	229	2345	4266	928	2454
	3,4	312,173	623,985	43,514	66,179	190	227	2330	4238	916	2422
**Sub-Saharan Africa**	1,2,5,6	1,517,327	3,025,939	212,901	322,443	942	1118	12,223	22,891	10,967	32,735
	3,4	1,449,400	2,889,696	203,532	308,103	901	1070	11,673	21,846	10,478	31,289
**Latin America & Caribbean**	1,2,5,6	286,031	571,793	39,855	60,626	174	208	7949	11,387	1163	3235
	3,4	297,851	595,385	41,511	63,136	182	216	8308	11,895	1204	3350
**East Asia & Pacific**	1,2,5,6	831,037	1,661,287	115,783	176,134	506	603	6386	10,512	1970	4811
	3,4	862,246	1,723,558	120,157	182,764	525	626	6626	10,902	2038	4980
**North America**	1,2,5,6	150,714	301,372	20,984	31,937	92	109	7568	16,919	9	16
	3,4	150,087	300,114	20,898	31,805	91	109	7538	16,851	9	16
**Global**	1,2,5,6	4,413,825	8,815,571	616,705	936,537	2709	3224	47,860	87,977	18,179	54,646
	3,4	4,408,757	8,804,622	616,192	935,583	2709	3223	48,057	88,105	17,833	53,619

The baseline cases (pre-vaccination) of Strep A disease burden (pharyngitis, impetigo, invasive disease, cellulitis, and rheumatic heart disease) is presented at the regional (United Nations regions) and global levels, based on 30 birth cohorts from year of vaccine introduction and for duration associated with the vaccination scenarios. Case numbers are split between those scenarios that have country-specific years of introduction (1,2,5,6) and those introduced in 2022 (3,4). The left column of case numbers relate to the 10-year durability assumption and the right column to 20-year durability of vaccine protection.

**Table 4:** Vaccine impact at the regional and global levels.

UN regions	Scenarios	Fully vaccinated individuals (millions)	Cases averted through vaccination (thousands) (range for scenarios 1-2 and 3-6)				
			Pharyngitis	Impetigo	Invasive disease	Cellulitis	Rheumatic heart disease
**South Asia**	1–2*	657	(578,344, 606,913)	(76,156, 80,716)	(291, 310)	(3880, 3992)	(1041, 1466)
	3–6	(381, 388)	(334,999, 357,905)	(44,117, 47,620)	(169, 183)	(2246, 2352)	(608, 870)
**Europe & Central Asia**	1–2*	226	(199,636, 209,585)	(26,231, 27,796)	(100, 106)	(2027, 2104)	(102, 120)
	3–6	(122, 124)	(107,661, 114,573)	(14,146, 15,197)	(54, 58)	(1115, 1171)	(53, 62)
**Middle East & North Africa**	1–2*	218	(192,160, 201,701)	(25,266, 26,776)	(96, 102)	(1253, 1258)	(348, 407)
	3–6	(121, 122)	(106,638, 112,917)	(14,025, 14,991)	(53, 57)	(693, 701)	(196, 232)
**Sub-Saharan Africa**	1–2*	918	(799,501, 838,027)	(105,671, 112,075)	(407, 433)	(5548, 5635)	(3635, 4633)
	3–6	(583, 607)	(505,846, 553,384)	(62,933, 74,061)	(258, 286)	(3562, 3777)	(2285, 3030)
**Latin America & Caribbean**	1–2*	184	(162,482, 170,555)	(21,361, 22,637)	(81, 87)	(3544, 3949)	(413, 501)
	3–6	(109, 113)	(95,898, 104,796)	(12,608, 13,913)	(48, 53)	(2091, 2435)	(244, 306)
**East Asia & Pacific**	1-2*	575	(507,378, 532,590)	(66,695, 70,679)	(254, 270)	(3261, 3354)	(763, 860)
	3-6	(317, 329)	(279,857, 304,513)	(36,790, 40,426)	(140, 155)	(1837, 1954)	(414, 483)
**North America**	1–2*	107	(94,334, 99,039)	(12,395, 13,134)	(47, 50)	(4145, 4506)	(4, 4)
	3–6	(58, 58)	(51,094, 53,879)	(6,714, 7,145)	(26, 27)	(2245, 2451)	(2, 2)
**Global**	1–2*	2886	(2,533,834, 2,658,410)	(333,775, 353,814)	(1277, 1359)	(23,657, 24,797)	(6306, 7991)
	3–6	(1690, 1741)	(1,481,995, 1,601,967)	(195,332, 213,353)	(748, 820)	(13,789, 14,843)	(3802, 4985)

The vaccine impact on cases averted is presented at the regional (United Nations regions) and global levels for different scenarios, based on the lifetime health impact of vaccination at birth for 30 birth cohorts from year of vaccine introduction on Strep A disease burden (pharyngitis, impetigo, invasive disease, cellulitis, and rheumatic heart disease).

^*^ Same number of fully vaccinated individuals for scenarios 1 and 2.

**Table 5:** Vaccine impact (deaths averted by vaccination at birth) at the regional and global levels.

UN regions	Scenarios	Fully vaccinated individuals (millions)	Deaths averted by vaccination at birth (thousands) (range for scenarios 1-2 and 3-6)	
			Invasive disease	Rheumatic heart disease
**South Asia**	1–2*	657	(18, 19)	(313, 440)
	3–6	(381, 388)	(10, 11)	(183, 261)
**Europe & Central Asia**	1–2*	226	(6, 7)	(30, 36)
	3–6	(122, 124)	(3, 4)	(16, 18)
**Middle East & North Africa**	1–2*	218	(6, 6)	(103, 121)
	3–6	(121, 122)	(3, 4)	(58, 69)
**Sub-Saharan Africa**	1–2*	918	(25, 27)	(1092, 1392)
	3–6	(583, 607)	(16, 18)	(686, 910)
**Latin America & Caribbean**	1–2*	184	(5, 5)	(122, 148)
	3–6	(109, 113)	(3, 3)	(72, 91)
**East Asia & Pacific**	1–2*	575	(16, 17)	(229, 258)
	3–6	(317, 329)	(9, 9)	(124, 145)
**North America**	1–2*	107	(3, 3)	(0.11, 0.11)
	3–6	(58, 58)	(2, 2)	(0.06, 0.06)
**Global**	1–2*	2886	(79, 83)	(1889, 2395)
	3–6	(1690, 1741)	(46, 50)	(1139, 1494)

The vaccine impact on deaths averted (in thousands) is presented at the regional (United Nations regions) and global levels for different scenarios, based on the lifetime health impact of vaccination administered at birth for 30 birth cohorts from year of vaccine introduction on Strep A disease burden (invasive disease and rheumatic heart disease).

^*^ Same number of fully vaccinated individuals for scenarios 1 and 2.

### Vaccine impact on Strep A cases

Globally, we estimated that vaccination at birth for 30 cohorts assuming country-specific vaccine introduction between 2022 and 2034 under scenario 1 could avert 2.5 billion cases of pharyngitis, 354 million cases of impetigo, 1.4 million cases of invasive disease, 24 million cases of cellulitis, and 6 million cases of RHD during the vaccinated individuals’ lifetime (see Table 4 for scenario 1). This averages to 82 million cases of pharyngitis, 11.8 million cases of impetigo, 45,000 cases of invasive disease, 805,000 cases of cellulitis, and 210,000 cases of RHD averted per birth cohort.

Regionally, we estimated vaccination impact in terms of total cases averted to be highest in Sub-Saharan Africa (see Table 4) for pharyngitis, impetigo, invasive disease, cellulitis, and RHD. By income level, vaccination impact on Strep A cases averted was estimated to be highest in lower-middle-income countries (see Fig. 2).

Vaccination impact in terms of cases averted per 1000 vaccinated individuals was highest in North America for cellulitis and in Sub-Saharan Africa for RHD (see Fig. 3). The vaccine impact metric of disease burden averted per 1000 vaccinated individuals remains the same for any vaccination coverage in each scenario, with the caveat that the Strep A vaccine impact model includes only the direct effects of vaccination and excludes indirect herd effects. As only the direct effect of vaccination was included, the estimated health benefits of Strep A vaccination are likely to be conservative.

### Vaccine impact on Strep A-related deaths

Globally, we estimated that vaccination at birth for 30 cohorts under scenario 1 could avert 1.9 million deaths resulting from RHD and 79,000 deaths from invasive disease (see Table 5). Over 1 million of these global RHD deaths averted are in Sub-Saharan Africa (see Table 5).

### Vaccine impact on DALYs

Nationally (see Fig. 4), we estimated the combined disability-adjusted life years (DALYs) per 1000 vaccinated individuals averted due to a Strep A vaccine. This averted burden was highest in Tonga and Sub-Saharan African countries.

### Vaccine coverage levels and year of introduction

The numbers of fully vaccinated individuals were higher for scenarios 1-2 due to higher levels of vaccine coverage assumed for many of the countries (see Supplementary Information) and hence a higher number of cases were averted in these scenarios compared with scenarios 3-6 (see Table 4). However, a delay in vaccine introduction occurs in scenarios 1–2 and 5–6 which leads to a corresponding delay in vaccination impact compared with scenarios 3–4 where Strep A vaccination was assumed to be introduced in all countries in 2022.

### Age of vaccination

Cases averted per 1000 fully vaccinated individuals were higher for pharyngitis and RHD when vaccination was administered to children aged 5 years compared with vaccination at birth (see Figs. 2, 3). This is because the pre-vaccination burden (cumulative incidence) of pharyngitis and RHD is higher in the 10–20 years after 5 years of age than the cumulative incidence in the 10–20 years after birth among the 30 birth cohorts of 2022–2051. Note that scenarios 1,3,5 assume full efficacy of vaccine-derived immunity for 10 years and scenarios 2,4,6 assume linear waning over 20 years.

Similarly, the combined DALYs averted resulting from a Strep A vaccine at the national level (see Fig. 4) in countries in Sub-Saharan Africa, South America, and the Caribbean were higher when vaccination was administered at 5 years of age in comparison to vaccination at birth, primarily due to the higher number of RHD cases averted per 1000 vaccinated individuals after 5 years of age.

## Discussion

We estimated the potential health impact of prospective Strep A vaccination at the global, regional, and national levels and by country-income category, using the Strep A vaccine impact model. Based on the WHO preferred product characteristics for Strep A vaccines^15^, we developed this static cohort model to estimate the lifetime health benefits among the vaccinated cohorts, with a focus on Strep A disease burden averted for pharyngitis, impetigo, invasive disease, cellulitis, and RHD. We analysed strategic scenarios for vaccination at birth and 5 years of age and projected that around one-third of the annual global burden of RHD can be potentially averted by prospective Strep A vaccination. Regionally, vaccination impact in terms of burden averted per fully vaccinated individual is highest in North America for cellulitis and in Sub-Saharan Africa for RHD, since the underlying disease burden per 100,000 population is relatively higher for cellulitis and RHD in North America and Sub-Saharan Africa respectively. By income level, total vaccine-avertable burdens for pharyngitis, impetigo, invasive disease, cellulitis, and RHD were highest in lower-middle-income countries, primarily due to the demographic effect of larger population sizes among the target ages for vaccination.

Our health impact estimates provide useful decision-making support for clinical development, interpretation of existing data, assessing needs for data collection to close critical evidence gaps, and policy decisions for prioritisation and implementation throughout the end-to-end continuum of discovery, development, and delivery of safe, effective, and affordable (prospective) Strep A vaccines. While the vaccine-avertable disease burden is an important public health priority, the high morbidity and mortality impact on the young adult population also translates into considerable productivity losses from an economic perspective^7,17^. This requires a broader global public health value proposition analysis to infer the end-to-end perspectives of vaccine development to uptake continuum^14,24–26^, and the global benefits of prospective Strep A vaccines are estimated to be highly positive^27^.

Our study has limitations. We estimated only the direct effect of Strep A vaccination and excluded the indirect effect on preventing pathogen transmission. Evidence on Strep A transmission modes would be valuable to develop a transmission dynamic model for estimating the total (direct and indirect) effects of vaccination. Such estimates would plausibly increase the impact of a Strep A vaccine, especially a vaccine administered in the first few years of life, given the peak incidences of severe invasive disease occurring in infants and older adults. Instead of endogenous modeling of transmission dynamics, the impact of indirect herd effects could be extrapolated by specifying a basic multiplier of the direct effect in the static cohort model, similar to the evaluation of *Haemophilus influenzae* type b, pneumococcal, and rotavirus vaccination^28^. However if Strep A vaccines significantly change the force of infection, then it may warrant a transmission dynamic model to assess the non-linear nature of Strep A infection dynamics^29^. There could be shifts in the median age of infection and Strep A disease burden beyond the duration of protection conferred by vaccination, as well as reduction in Strep A burden among non-vaccinated individuals due to herd immunity^30^.

Evidence on the natural history of disease dynamics would be beneficial to simulate disease progression, such as the progression from acute rheumatic fever to RHD. We estimated vaccination impact on Strep A burden averted for pharyngitis, impetigo, invasive disease, cellulitis, and RHD but excluded the immune-mediated sequelae of acute rheumatic fever and acute post-streptococcal glomerulonephritis and the etiological pathway between Strep A infection and those diseases. While the exclusion of ARF and APSGN underestimates the impact of a vaccine that prevents those diseases, excluding the etiological pathway between infection and immune-mediated sequelae, including RHD, may overestimate the impact of vaccination at 5 years of age and underestimate the impact of infant vaccination. In our model, we assumed that vaccination in 5-year-old children would prevent incident cases of RHD occurring at age of 5 years. However, some proportion of those cases may be averted by an infant vaccination schedule that prevents the infections preceding ARF and RHD. Further epidemiology or immunology studies are required to model this scenario.

The vaccine impact model can be extended to include other Strep A disease states and sequelae to estimate the additional health benefits of vaccination in averting morbidity and mortality attributable to these conditions. The health benefits of vaccination in reducing Strep A infections could lower corresponding antibiotic use (to treat Strep A infections), and the model could capture this feature with the availability of quality observational data on the prescription patterns and levels of antibiotic use in treating Strep A infections. The WHO views vaccination as having additional value in reducing antibiotic consumption^31^. Reducing antibiotic consumption may feasibly lower the risk and rate of antibiotic resistance in Strep A and on off-target, or bystander, pathogens, such as pneumococcus. It may also reduce the risk of microbiome disruption, which is hypothesised to lead to chronic health conditions^32^.

The vaccine impact projections are based on a hypothetical vaccine that meets the WHO preferred product characteristics^15^, and Strep A vaccines that attain licensure may have varied characteristics. The vaccine coverage assumptions are based on past coverage trends for *Haemophilus influenzae* type B vaccine (Hib3) or the third dose of diphtheria, tetanus, and pertussis vaccine, where Hib3 coverage values were unavailable, and coverage and scale-up of future Strep A vaccines may differ. Also, assumptions for the duration of protection and waning dynamics of vaccine-derived immunity may differ from future evidence generated from clinical trials and effectiveness studies.

To our knowledge, our model is the first to predict the vaccine impact for multiple Strep A diseases for more than 200 countries. Two other studies have developed similar models, but they were calibrated to data from Australia and New Zealand^17,18^. Both countries have endemic RHD in their marginalized, Indigenous populations, and advanced disease surveillance systems. The models developed for those countries included the onset of ARF and the subsequent risk of progression to RHD. However, data on the risks of ARF and progression to RHD are limited for other countries, and consequently, only the incidence of RHD was modeled in this study. Better epidemiological data will facilitate improved predictions of future disease burden and impact of vaccines.

In conclusion, based on the WHO preferred product characteristics for Strep A vaccines, we developed a Strep A vaccine impact model to estimate the health benefits of vaccination in terms of disease burden averted for pharyngitis, impetigo, invasive disease, cellulitis, and RHD at the global, regional, national, and country-income levels. The health impact estimates of Strep A vaccination are useful inputs for generating economic impact estimates based on cost-effectiveness analysis and for societal impact estimates based on benefit-cost analysis, thereby collating useful pieces of evidence towards the full value of vaccine assessment^24,33^ for Strep A vaccines.

## Methods

### Strep A vaccine impact model

We developed a static cohort model to estimate the lifetime health benefits of vaccination for 30 cohorts from the year of vaccine introduction (2022-2034) on Strep A disease burden (pharyngitis, impetigo, invasive disease, cellulitis, and RHD) in terms of episodes/cases, deaths, and disability-adjusted life years (DALYs) averted by vaccination (see Fig. 1). The reduction in disease burden is in direct proportion to vaccine efficacy, vaccine coverage, and vaccine-derived immunity. However, this assumption takes into account only the direct effect and excludes indirect herd effect and the non-linear transmission dynamics of Strep A infections. Thereby, for ages that lie within the duration of protection since the time of vaccination:

$$\begin{array}{rcl}{\rm{Strep}}\,{\rm{A}}\,burden\,{\rm{averted}}\,{\rm{at}}\,{\rm{age}}\,i & = & {\rm{Strep}}\,{\rm{A}}\,burden\,{\rm{at}}\,{\rm{age}}\,i\,{\rm{pre}}\hbox{-}{\rm{vaccination}} \\ \times & & {\rm{vaccine}}\,{\rm{efficacy}} \\ \times & & {\rm{vaccine}}\,{\rm{coverage}}\end{array}$$

The pre-vaccination disease burden is based on country- and age-specific incidence rates for cellulitis and RHD and global age-specific prevalence for impetigo from the 2019 Global Burden of Disease (GBD) study^23^. For pharyngitis and invasive disease, the pre-vaccination disease burden is based on global age-specific rates from systematic reviews conducted as part of the Strep A Vaccine Global Consortium (SAVAC) project. Mortality risk was limited to 28 days from hospitalisation for invasive disease and to 10 years from disease onset for RHD. Country- and age-specific rates of Strep A burden were assumed to remain constant in the future. We did not include APSGN and ARF in our analysis due to data limitations on prevalent burden estimates.

Demographic estimates for the country, year and age-specific population, all-cause mortality rates, and remaining life expectancy are based on the 2019 United Nations World Population Prospects^34^. The model uses non-sex specific projected (2020–2100) interpolated age-specific (0–99 years) population and age-grouped (covering the same age range) all-cause mortality probabilities and remaining life expectancy estimates. Age groups for all-cause mortality and life expectancy are in 5-year bands, apart from 0 to 4 years (age 0 is in a separate group), assuming uniformity within groups for mortality and remaining life expectancy. Any projection of lifetime burden that went beyond 2100 assumed the same population, all-cause mortality, and remaining life expectancy values as for 2100. The country- and age-specific population numbers were used to estimate the population at age of vaccination, and then all-cause mortality probabilities were used to estimate the modeled population at each age over a cohort’s lifetime.

Disability weights used for the calculation of years lived with disability are from the GBD study^35^, and YLD (years lived with disability) was attributed to the years of prevalence. The duration for pharyngitis, impetigo, invasive disease, and cellulitis was estimated to be 5 days, 15.5 days, 10 days, and 16.4 days respectively, based on the GBD reported prevalence divided by incidence^23^. The duration for RHD was assumed to be the remaining life expectancy from the onset of the condition.

The vaccine efficacy assumptions are based on the WHO preferred product characteristics for Strep A vaccine, as shown in Table 1^15^. The waning dynamics of vaccine-derived immunity were modeled in two ways: (i) vaccine-induced immune protection at maximum efficacy for 10 years and null thereafter and (ii) waning linearly with an annual reduction in efficacy equivalent to 5% of maximum efficacy for 20 years and null thereafter (i.e., waning to 50% of maximum efficacy after 10 years). The year of vaccine introduction was assumed to be 2022 or country-specific ranging from 2022 to 2034, with initial coverage at 10% of maximum coverage. The vaccine coverage was assumed to scale up linearly during the first 10 years after introduction to reach either a maximum of 50% coverage for all countries or a country-specific coverage ranging from 9% to 99%. Country-specific coverage values and year of introduction (see Supplementary Information) are based on past trends for Hib3 or the third dose of diphtheria, tetanus, and pertussis vaccine where Hib3 values were unavailable.

Using the model, we analysed six potential scenarios for varied years of vaccine introduction, coverage, and waning dynamics (see Table 2), with vaccination at birth or 5 years of age. We modeled these scenarios for 183 countries (see Supplementary Information) and aggregated results at the global, regional, and country-income levels.

While these vaccination scenarios are hypothetical, the pragmatic vaccination scenarios will become clear during the vaccine introduction discussions at the national (including national immunization technical advisory groups), regional (including regional immunization technical advisory groups), and global (including WHO Strategic Advisory Group of Experts on immunization) levels. However, the vaccine impact metric of disease burden averted per fully vaccinated individual remains the same for any vaccination coverage for a given scenario, with the caveat that the Strep A vaccine impact model includes only the direct effects of vaccination and excludes indirect herd effects.

### Reporting summary

Further information on research design is available in the Nature Research Reporting Summary linked to this article.

## Supplementary information

Supplementary Information

REPORTING SUMMARY

## Data Availability

The data used in the model are publicly available and can be accessed through the program code available on *github*.

## Code availability

The program code and data for the vaccine impact model is available as an R package on *github* along with the code for the accompanying R Shiny web application; (https://github.com/fionagi/GASImpactModel) and (https://github.com/fionagi/GASImpactModel_App).

## References

[1] Carapetis JR, Steer AC, Mulholland EK, Weber M (2005). The global burden of group A streptococcal diseases. Lancet Infect. Dis..

[2] Watkins DA (2017). Global, regional, and national burden of rheumatic heart disease, 1990–2015. N. Engl. J. Med..

[3] Ralph AP, Carapetis JR (2012). Group A Streptococcal diseases and their global burden. Microbiol Immunol..

[4] Sims Sanyahumbi, A., Colquhoun, S., Wyber, R. & Carapetis, J. R. in *Streptococcus pyogenes: Basic Biology to Clinical Manifestations* (eds. Ferretti, J. J., Stevens, D. L. & Fischetti, V. A.) (University of Oklahoma Health Sciences Center, 2016). 26866208

[5] Wang D (2013). Stroke and rheumatic heart disease: a systematic review of observational studies. Clin. Neurol. Neurosurg..

[6] Karthikeyan G, Mayosi BM (2009). Is primary prevention of rheumatic fever the missing link in the control of rheumatic heart disease in Africa?. Circulation.

[7] Cannon J, Bessarab DC, Wyber R, Katzenellenbogen JM (2019). Public health and economic perspectives on acute rheumatic fever and rheumatic heart disease. Med. J. Aust..

[8] Nelson GE (2016). Epidemiology of invasive group A Streptococcal infections in the United States, 2005–2012. Clin. Infect. Dis..

[9] Raff AB, Kroshinsky D (2016). Cellulitis: a review. JAMA.

[10] Cannon J, Rajakaruna G, Dyer J, Carapetis J, Manning L (2018). Severe lower limb cellulitis: defining the epidemiology and risk factors for primary episodes in a population-based case-control study. Clin. Microbiol. Infect..

[11] Fleming-Dutra KE (2016). Prevalence of inappropriate antibiotic prescriptions among US ambulatory care visits, 2010–2011. JAMA.

[12] Miller, K. M. et al. Antibiotic consumption for sore throat and the potential effect of a vaccine against group A *Streptococcus*: a systematic review and modelling study. *SSRN J.*10.2139/ssrn.4344471 (2023). 10.1016/j.ebiom.2023.104864 PMC10663680 37950997

[13] Barth DD (2021). Modes of transmission and attack rates of group A Streptococcal infection: a protocol for a systematic review and meta-analysis. Syst. Rev..

[14] Vekemans J (2019). The path to group A Streptococcus vaccines: World Health Organization research and development technology roadmap and preferred product characteristics. Clin. Infect. Dis..

[15] World Health Organization. *WHO Preferred Product Characteristics for Group A Streptococcus Vaccines*. https://apps.who.int/iris/handle/10665/279142 (2018).

[16] Coates MM (2021). An investment case for the prevention and management of rheumatic heart disease in the African Union 2021-30: a modelling study. Lancet Glob. Health.

[17] Cannon JW (2018). An economic case for a vaccine to prevent group A Streptococcus skin infections. Vaccine.

[18] Cannon JW (2021). The economic and health burdens of diseases caused by group A Streptococcus in New Zealand. Int. J. Infect. Dis..

[19] R Core Team. *R: A Language and Environment for Statistical Computing* (R Foundation for Statistical Computing, 2019).

[20] Echeverria-Londono S (2021). How can the public health impact of vaccination be estimated?. BMC Public Health.

[21] Miller KM (2022). The global burden of sore throat and group A Streptococcus pharyngitis: a systematic review and meta-analysis. EClinicalMedicine.

[22] Carapetis, J. R., Cannon, J. W. & Moore, H. C. *A Strategic Approach to Understanding Strep A Disease Burden* (SAVAC, 2022).

[23] GBD 2019 Diseases and Injuries Collaborators. (2020). Global burden of 369 diseases and injuries in 204 countries and territories, 1990-2019: a systematic analysis for the Global Burden of Disease Study 2019. Lancet.

[24] Hutubessy, R. C. W. et al. The full value of vaccine assessments (FVVA): A framework to assess and communicate the value of vaccines for investment and introduction decision making. *SSRN J.*10.2139/ssrn.3841999 (2021). 10.1186/s12916-023-02929-0 PMC10318807 37400797

[25] Steer AC (2016). Status of research and development of vaccines for Streptococcus pyogenes. Vaccine.

[26] Steer AC, Dale JB, Carapetis JR (2013). Progress toward a global group a Streptococcal vaccine. Pediatr. Infect. Dis. J..

[27] Cadarette, D., Ferranna, M., Cannon, J. & Bloom, D. *The Full Health, Economic, and Social Benefits of Strep A Vaccines* (SAVAC, 2022). 10.1038/s41541-023-00758-z PMC10616198 37903813

[28] Clark A (2013). TRIVAC decision-support model for evaluating the cost-effectiveness of Haemophilus influenzae type b, pneumococcal and rotavirus vaccination. Vaccine.

[29] Jit M, Brisson M (2011). Modelling the epidemiology of infectious diseases for decision analysis: a primer. Pharmacoeconomics.

[30] Brisson M, Edmunds WJ (2003). Economic evaluation of vaccination programs: the impact of herd-immunity. Med. Decis. Mak..

[31] WHO. *Bacterial Vaccines in Clinical and Preclinical Development 2021: An Overview and Analysis*. https://www.who.int/publications/i/item/9789240052451 (2022).

[32] Korpela K (2016). Intestinal microbiome is related to lifetime antibiotic use in Finnish pre-school children. Nat. Commun..

[33] Cadarette, D., Ferranna, M., Cannon, J. & Bloom, D. The full health, economic, and social benefits of Strep A vaccines. https://savac.ivi.int/documents/6.%20The%20full%20health%20economic%20social%20benefits%20of%20vaccination_conceptual%20framework%20and%20application%20to%20Strep%20A%20vaccines_Dan%20Cadarette.pdf (SAVAC, 2022). 10.1038/s41541-023-00758-z PMC10616198 37903813

[34] UNDP. *World Population Prospects—Population Division—United Nations*. https://population.un.org/wpp/ (2019).

[35] Salomon JA (2015). Disability weights for the global burden of disease 2013 study. Lancet Glob. Health.

