# Author Correction: Modeling the potential health impact of prospective Strep A vaccines

**DOI:** 10.1038/s41541-023-00704-z

**Published:** 2023-07-15

**Authors:** Fiona Giannini, Jeffrey W. Cannon, Daniel Cadarette, David E. Bloom, Hannah C. Moore, Jonathan Carapetis, Kaja Abbas

**Affiliations:** 1 Wesfarmers Centre for Vaccines and Infectious Diseases, Telethon Kids Institute, University of Western Australia, Perth, Australia; 2 Harvard T.H. Chan School of Public Health, Boston, USA; 3 School of Population Health, Curtin University, Perth, Australia; 4 Perth Children’s Hospital, Perth, Australia; 5 London School of Hygiene & Tropical Medicine, London, UK

**Keywords:** Diseases, Epidemiology, Epidemiology

Correction to: *npj Vaccines* 10.1038/s41541-023-00668-0, published online 10 June 2023

In the original version of this Article, additional boxes were shown in Figure 1 that should have been removed for publication. Figure 1 has been corrected in the PDF and HTML version of this Article. In addition, a few typographical errors were corrected and higher resolution image files used for Figures 2–4.

