# Supplementary material for: Modeling the potential health impact of prospective Strep A vaccines
Source: NPJ Vaccines. 2023 Jun 10;8:90. doi: 10.1038/s41541-023-00668-0 (PMC10256967; doi:10.1038/s41541-023-00668-0)

Supplementary Information

Supplementary Table 1. Vaccine impact (cases averted by vaccination at 5 years of age) at the regional and global levels. The vaccine impact on cases averted is presented at the regional (United Nations regions) and global levels for different scenarios, based on the lifetime health impact of vaccination administered at 5 years of age for 30 vaccinated cohorts on Strep A disease burden (pharyngitis, impetigo, invasive disease, cellulitis, and rheumatic heart disease).

UN regions	Scenarios	Fully vaccinated individuals (millions)	**Cases averted by vaccination at 5 years of age** (thousands) (range for scenarios 1-2 and 3-6)				
			Pharyngitis	Impetigo	Invasive disease	Cellulitis	Rheumatic heart disease
South Asia	1-2*	688	(941,532, 1,214,642)	(63,183, 72,453)	(72, 80)	(3,921, 4,118)	(2,077, 2,480)
	3-6	(398, 405)	(544,948, 715,582)	(36,569, 42,684)	(42, 47)	(2,268, 2,424)	(1,211, 1,469)
Europe & Central Asia	1-2*	230	(314,454, 405,725)	(21,104, 24,199)	(24, 27)	(1,772, 1,934)	(160, 165)
	3-6	(124, 126)	(169,651, 222,327)	(11,386, 13,260)	(13, 15)	(981, 1,085)	(83, 86)
Middle East & North Africa	1-2*	217	(297,569, 383,917)	(19,970, 22,899)	(23, 25)	(1,160, 1,200)	(526, 556)
	3-6	(121, 122)	(165,632, 215,138)	(11,115, 12,832)	(13, 14)	(644, 670)	(297, 317)
Sub-Saharan Africa	1-2*	884	(1,206,793, 1,556,228)	(80,963, 92,852)	(92, 102)	(5,070, 5,256)	(6,419, 6,501)
	3-6	(558, 585)	(761,012, 1,028,952)	(51,052, 61,395)	(58, 68)	(3,242, 3,527)	(4,004, 4,248)
Latin America & Caribbean	1-2*	191	(262,127, 338,193)	(17,591, 20,172)	(20, 22)	(3,023, 3,276)	(710, 728)
	3-6	(113, 118)	(154,736, 208,235)	(10,384, 12,420)	(12, 14)	(1,782, 2,022)	(419, 446)
East Asia & Pacific	1-2*	592	(811,126, 1,046,509)	(54,436, 62,420)	(62, 69)	(2,549, 2,602)	(1,154, 1,162)
	3-6	(327, 340)	(447,406, 599,972)	(30,026, 35,786)	(34, 39)	(1,435, 1,518)	(626, 654)
North America	1-2*	108	(148,060, 191,037)	(9,937, 11,394)	(11, 13)	(4,639, 5,105)	(2, 3)
	3-6	(58, 59)	(80,100, 103,928)	(5,376, 6,199)	(6, 7)	(2,509, 2,777)	(1, 1)
Global	1-2*	2,911	(3,981,660, 5,136,251)	(267,184, 306,388)	(305, 338)	(22,134, 23,493)	(11,049, 11,594)
	3-6	(1,699, 1,754)	(2,323,484, 3,094,135)	(155,909, 184,577)	(178, 204)	(12,861, 14,022)	(6,641, 7,222)

* Same number of fully vaccinated individuals for scenarios 1 and 2.

Supplementary Table 2. Vaccine impact (DALYs averted by vaccination at birth) at the regional and global levels. The vaccine impact on DALYs averted (in thousands) is presented at the regional (United Nations regions) and global levels for different scenarios, based on the lifetime health impact of vaccination administered at birth for 30 vaccinated cohorts on Strep A disease burden (pharyngitis, impetigo, invasive disease, cellulitis, and rheumatic heart disease).

**UN regions**	**Scenarios**	**Fully vaccinated individuals (millions)**	**DALYs averted by vaccination at birth** (thousands) (range for scenarios 1-2 and 3-6)				
			**Pharyngitis**	**Impetigo**	**Invasive disease**	**Cellulitis**	**Rheumatic heart disease**
South Asia	1-2*	657	(206, 216)	(19, 21)	(1,321, 1,400)	(9, 9)	(24,398, 32,854)
	3-6	(381, 388)	(119, 127)	(11, 12)	(764, 820)	(5, 5)	(14,229, 19,354)
Europe & Central Asia	1-2*	226	(71, 75)	(7, 7)	(495, 525)	(5, 5)	(2,387, 2,720)
	3-6	(122, 124)	(38, 41)	(4, 4)	(267, 284)	(3, 3)	(1,233, 1,413)
Middle East & North Africa	1-2*	218	(68, 72)	(6, 7)	(458, 485)	(3, 3)	(8,394, 9,507)
	3-6	(121, 122)	(38, 40)	(4, 4)	(251, 271)	(2, 2)	(4,678, 5,410)
Sub-Saharan Africa	1-2*	918	(285, 298)	(27, 29)	(1,710, 1,813)	(13, 13)	(79,908, 98,237)
	3-6	(583, 607)	(180, 197)	(17, 19)	(1,068, 1,191)	(8, 9)	(49,637, 63,933)
Latin America & Caribbean	1-2*	184	(58, 61)	(5, 6)	(398, 422)	(8, 9)	(10,341, 12,141)
	3-6	(109, 113)	(34, 37)	(3, 4)	(235, 256)	(5, 6)	(6,095, 7,338)
East Asia & Pacific	1-2*	575	(181, 190)	(17, 18)	(1,240, 1,314)	(7, 8)	(19,143, 21,000)
	3-6	(317, 329)	(100, 108)	(9, 10)	(682, 739)	(4, 4)	(10,356, 11,603)
North America	1-2*	107	(34, 35)	(3, 3)	(239, 253)	(9, 10)	(22, 22)
	3-6	(58, 58)	(18, 19)	(2, 2)	(128, 138)	(5, 6)	(12, 12)
Global	1-2*	2,886	(902, 946)	(85, 90)	(5,862, 6,212)	(54, 57)	(144,593, 176,481)
	3-6	(1,690, 1,741)	(527, 570)	(50, 54)	(3,396, 3,699)	(32, 34)	(86,241, 109,064)

*Same number of fully vaccinated individuals for scenarios 1 and 2.

Supplementary Table 3. Vaccine impact (DALYs averted by vaccination at 5 years of age) at the regional and global levels. The vaccine impact on DALYs averted (in thousands) is presented at the regional (United Nations regions) and global levels for different scenarios, based on the lifetime health impact of vaccination administered at 5 years of age for 30 vaccinated cohorts on Strep A disease burden (pharyngitis, impetigo, invasive disease, cellulitis, and rheumatic heart disease).

UN regions	Scenarios	Fully vaccinated individuals (millions)	**DALYs averted by vaccination at 5 years of age** (thousands) (range for scenarios 1-2 and 3-6)				
			**Pharyngitis**	**Impetigo**	**Invasive disease**	**Cellulitis**	**Rheumatic heart disease**
South Asia	1-2*	688	(335, 432)	(16, 18)	(366, 403)	(9, 9)	(45,824, 52,767)
	3-6	(398, 405)	(194, 255)	(9, 11)	(212, 236)	(5, 6)	(26,687, 31,032)
Europe & Central Asia	1-2*	230	(112, 144)	(5, 6)	(134, 148)	(4, 4)	(3,563, 3,570)
	3-6	(124, 126)	(60, 79)	(3, 3)	(72, 80)	(2, 2)	(1,842, 1,853)
Middle East & North Africa	1-2*	217	(106, 137)	(5, 6)	(121, 134)	(3, 3)	(12,008, 12,368)
	3-6	(121, 122)	(59, 77)	(3, 3)	(67, 75)	(1, 2)	(6,702, 7,049)
Sub-Saharan Africa	1-2*	884	(430, 554)	(21, 24)	(438, 481)	(12, 12)	(131,948, 133,244)
	3-6	(558, 585)	(271, 366)	(13, 16)	(273, 317)	(7, 8)	(81,343, 86,674)
Latin America & Caribbean	1-2*	191	(93, 120)	(4, 5)	(110, 122)	(7, 8)	(16,831, 16,905)
	3-6	(113, 118)	(55, 74)	(3, 3)	(65, 74)	(4, 5)	(9,929, 10,252)
East Asia & Pacific	1-2*	592	(289, 372)	(14, 16)	(339, 375)	(6, 6)	(27,130, 27,490)
	3-6	(327, 340)	(159, 214)	(8, 9)	(186, 211)	(3, 3)	(14,657, 15,220)
North America	1-2*	108	(53, 68)	(3, 3)	(64, 71)	(11, 12)	(13, 15)
	3-6	(58, 59)	(29, 37)	(1, 2)	(34, 39)	(6, 6)	(7, 8)
Global	1-2*	2,911	(1,417, 1,828)	(68, 78)	(1,573, 1,733)	(51, 54)	(237,317, 246,359)
	3-6	(1,699, 1,754)	(827, 1,101)	(40, 47)	(909, 1,030)	(29, 32)	(141,167, 152,088)

* Same number of fully vaccinated individuals for scenarios 1 and 2.

Supplementary Table 4. Vaccine impact (deaths averted by vaccination at 5 years of age) at the regional and global levels. The vaccine impact on deaths averted (in thousands) is presented at the regional (United Nations regions) and global levels for different scenarios, based on the lifetime health impact of vaccination administered at 5 years of age for 30 vaccinated cohorts on Strep A disease burden (invasive disease and rheumatic heart disease).

UN regions	Scenarios	Fully vaccinated individuals (millions)	Deaths averted by vaccination at 5 years of age (thousands) (range for scenarios 1-2 and 3-6)	
			Invasive disease	Rheumatic heart disease
South Asia	1-2*	688	(5, 6)	(624, 745)
	3-6	(398, 405)	(3, 4)	(364, 441)
Europe & Central Asia	1-2*	230	(2, 2)	(48, 49)
	3-6	(124, 126)	(1, 1)	(25, 25)
Middle East & North Africa	1-2*	217	(2, 2)	(156, 165)
	3-6	(121, 122)	(1, 1)	(88, 94)
Sub-Saharan Africa	1-2*	884	(7, 8)	(1,928, 1,953)
	3-6	(558, 585)	(4, 5)	(1,203, 1,276)
Latin America & Caribbean	1-2*	191	(1, 2)	(210, 216)
	3-6	(113, 118)	(1, 1)	(124, 132)
East Asia & Pacific	1-2*	592	(5, 5)	(346, 349)
	3-6	(327, 340)	(3, 3)	(188, 196)
North America	1-2*	108	(1, 1)	(0.07, 0.08)
	3-6	(58, 59)	(0.45, 0.52)	(0.04, 0.04)
Global	1-2*	2,911	(23, 26)	(3,313, 3,476)
	3-6	(1,699, 1,754)	(13, 15)	(1,992, 2,166)

* Same number of fully vaccinated individuals for scenarios 1 and 2.

Supplementary Table 5. Country-specific coverage and year of introduction for Strep A vaccination.

**Country**	**Vaccine coverage** (%)	**Vaccine introduction** (year)
Afghanistan	66	2030
Albania	99	2025
Algeria	91	2026
Angola	59	2027
Antigua and Barbuda	95	2027
Argentina	86	2027
Armenia	92	2022
Australia	94	2025
Austria	85	2030
Azerbaijan	95	2026
Bahamas, The	90	2027
Bahrain	99	2027
Bangladesh	98	2025
Barbados	95	2026
Belarus	9	2030
Belgium	97	2027
Belize	96	2028
Benin	76	2026
Bhutan	97	2028
Bolivia	83	2027
Bosnia and Herzegovina	62	2032
Botswana	95	2022
Brazil	83	2027
Brunei Darussalam	99	2030
Bulgaria	92	2027
Burkina Faso	91	2022
Burundi	90	2022
Cabo Verde	99	2025
Cambodia	92	2026
Cameroon	79	2027
Canada	91	2025
Central African Republic	47	2028
Chad	41	2030

Chile	95	2029
China	99	2028
Colombia	92	2027
Comoros	91	2025
Congo, Dem. Rep.	81	2028
Congo, Rep.	75	2025
Costa Rica	94	2025
Côte d'Ivoire	82	2025
Croatia	94	2030
Cuba	99	2028
Cyprus	97	2029
Czech Republic / Czechia	94	2030
Denmark	97	2029
Djibouti	84	2025
Dominican Republic	90	2027
Ecuador	85	2027
Egypt, Arab Rep.	95	2028
El Salvador	81	2027
Equatorial Guinea	25	2030
Eritrea	95	2022
Estonia	92	2027
Eswatini	90	2022
Ethiopia	72	2027
Fiji	99	2022
Finland	91	2027
France	95	2029
Gabon	70	2028
Gambia, The	93	2025
Georgia	93	2025
Germany	92	2027
Ghana	97	2022
Greece	99	2027
Grenada	96	2028
Guatemala	86	2027
Guinea	45	2030
Guinea-Bissau	88	2025

Guyana	95	2025
Haiti	64	2028
Honduras	90	2027
Hungary	99	2029
Iceland	91	2029
India	89	2025
Indonesia	79	2032
Iran, Islamic Rep.	99	2027
Iraq	84	2027
Ireland	94	2027
Israel	98	2027
Italy	94	2027
Jamaica	98	2027
Japan	99	2029
Jordan	96	2031
Kazakhstan	98	2029
Kenya	92	2022
Kiribati	95	2022
Korea, Dem. People's Rep.	97	2028
Korea, Rep.	98	2028
Kuwait	99	2027
Kyrgyz Republic	92	2025
Lao PDR	68	2028
Latvia	96	2027
Lebanon	85	2031
Lesotho	93	2025
Liberia	84	2025
Libya	97	2027
Lithuania	92	2027
Luxembourg	99	2027
Madagascar	75	2025
Malawi	92	2022
Malaysia	99	2028
Maldives	99	2028
Mali	71	2025
Malta	97	2030

Mauritania	81	2025
Mauritius	97	2025
Mexico	88	2030
Micronesia, Fed. Sts.	59	2025
Moldova	92	2027
Mongolia	99	2026
Montenegro	87	2033
Morocco	99	2025
Mozambique	80	2025
Myanmar	91	2026
Namibia	89	2025
Nepal	91	2026
Netherlands	93	2029
New Zealand	92	2025
Nicaragua	98	2025
Niger	89	2025
Nigeria	57	2028
North Macedonia	91	2030
Norway	96	2027
Oman	99	2029
Pakistan	75	2027
Panama	88	2027
Papua New Guinea	61	2027
Paraguay	88	2027
Peru	84	2027
Philippines	65	2032
Poland	95	2029
Portugal	99	2029
Qatar	98	2027
Romania	86	2032
Russian Federation	97	2030
Rwanda	97	2022
Samoa	34	2030
São Tomé and Principe	95	2022
Saudi Arabia	96	2027
Senegal	82	2022

Serbia	96	2029
Seychelles	99	2025
Sierra Leone	90	2022
Singapore	96	2029
Slovak Republic / Slovakia	96	2029
Slovenia	93	2029
Solomon Islands	85	2024
Somalia	42	2030
South Africa	74	2024
South Sudan	49	2030
Spain	94	2029
Sri Lanka	99	2030
St. Lucia	95	2027
St. Vincent and the Grenadines	97	2028
Sudan	93	2025
Suriname	95	2030
Sweden	97	2029
Switzerland	95	2029
Syrian Arab Republic	48	2033
Tajikistan	96	2026
Tanzania	98	2025
Thailand	97	2030
Timor-Leste	83	2030
Togo	88	2025
Tonga	81	2024
Trinidad and Tobago	99	2026
Tunisia	97	2030
Turkey	98	2029
Turkmenistan	99	2026
Uganda	93	2022
Ukraine	39	2033
United Arab Emirates	99	2025
United Kingdom	94	2026
United States	92	2027
Uruguay	91	2029
Uzbekistan	98	2025

Vanuatu	85	2025
Venezuela, RB	60	2034
Vietnam	75	2033
Yemen, Rep.	65	2027
Zambia	90	2025
Zimbabwe	89	2022

Supplementary Figure 1. Vaccine impact (DALYs averted) at the country-income level. The vaccine impact on DALYs averted (in thousands) is stratified by income levels of countries (World Bank income classification), based on the lifetime health impact of vaccination at birth or 5 years of age for 30 vaccinated cohorts on Strep A disease burden (pharyngitis, impetigo, invasive disease, cellulitis, and rheumatic heart disease). The vertical bars show the estimates for scenario 1, and the error bars show the range across scenarios 1-6. Note the differences in scale between the left panel (pharyngitis, impetigo and cellulitis) and the right panel (invasive and rheumatic heart disease).

Vaccination at birth

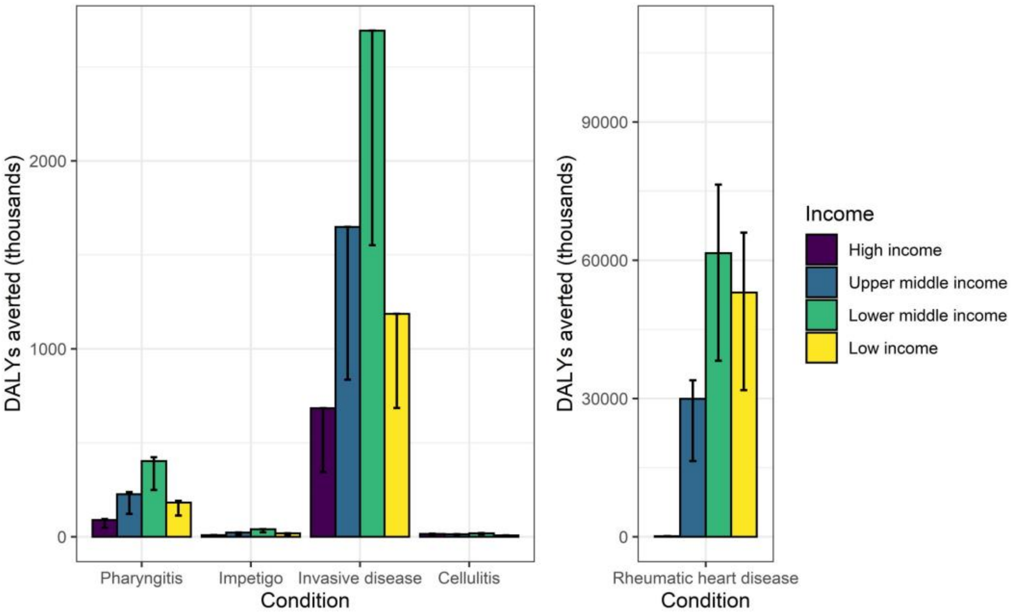

Vaccination at 5 years of age

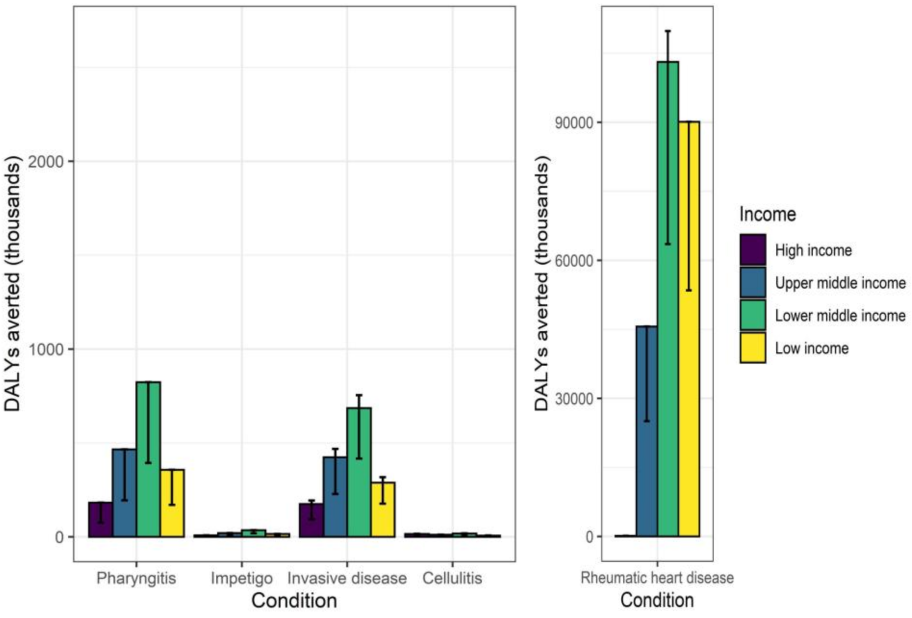

Supplementary Figure 2. Vaccine impact (deaths averted) at the country-income level. The vaccine impact on deaths averted (in thousands) is stratified by income levels of countries (World Bank income classification), based on the lifetime health impact of vaccination at birth or 5 years of age for 30 vaccinated cohorts on Strep A disease burden (invasive disease and rheumatic heart disease). The vertical bars show the estimates for scenario 1, and the error bars show the range across scenarios 1-6. Note the differences in scale between the left panel (invasive disease) and the right panel (rheumatic heart disease). For rheumatic heart disease in high-income countries, the range of values for age 0 is (0.3, 0.6) and for age 5 (0.4, 0.7).

Vaccination at birth

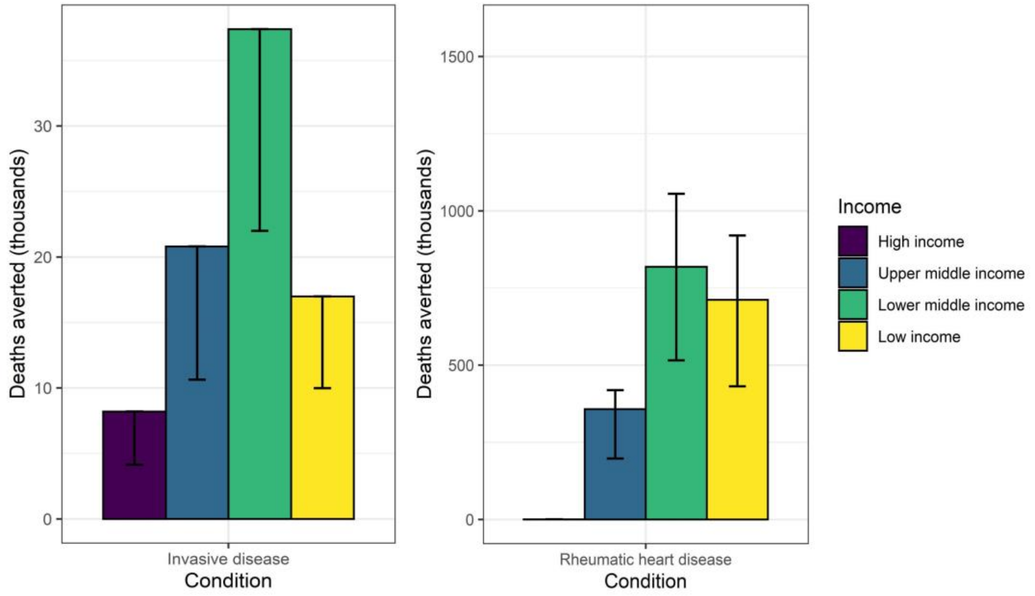

Vaccination at 5 years of age

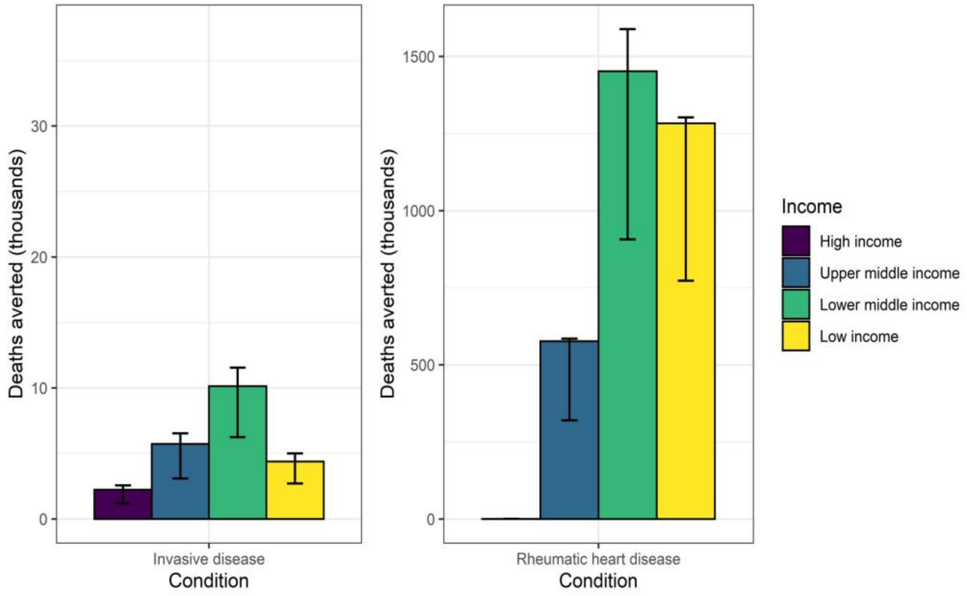

Supplementary Figure 3. Vaccine impact (DALYs averted per 1,000 fully vaccinated individuals) at the regional and global levels. The vaccine impact on DALYs averted per 1,000 fully vaccinated individuals is stratified at the regional (United Nations regions) and global levels for different scenarios (estimate for scenario 1 and range across the six scenarios), based on the lifetime health impact of vaccination at birth or 5 years of age for 30 vaccinated cohorts on Strep A disease burden (pharyngitis, impetigo, invasive disease, cellulitis, and rheumatic heart disease). The vertical bars show the estimates for scenarios 1, 3, and 5 (which are equal), and the error bars show the estimates for scenarios 2, 4, and 6 (which are equal).

Vaccination at birth

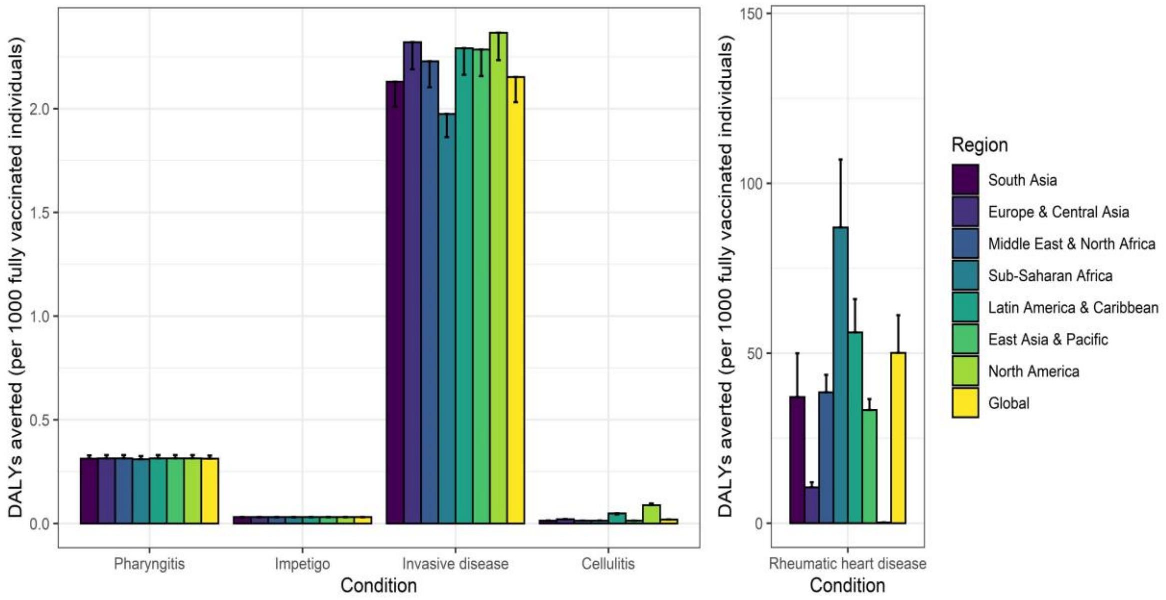

Vaccination at 5 years of age

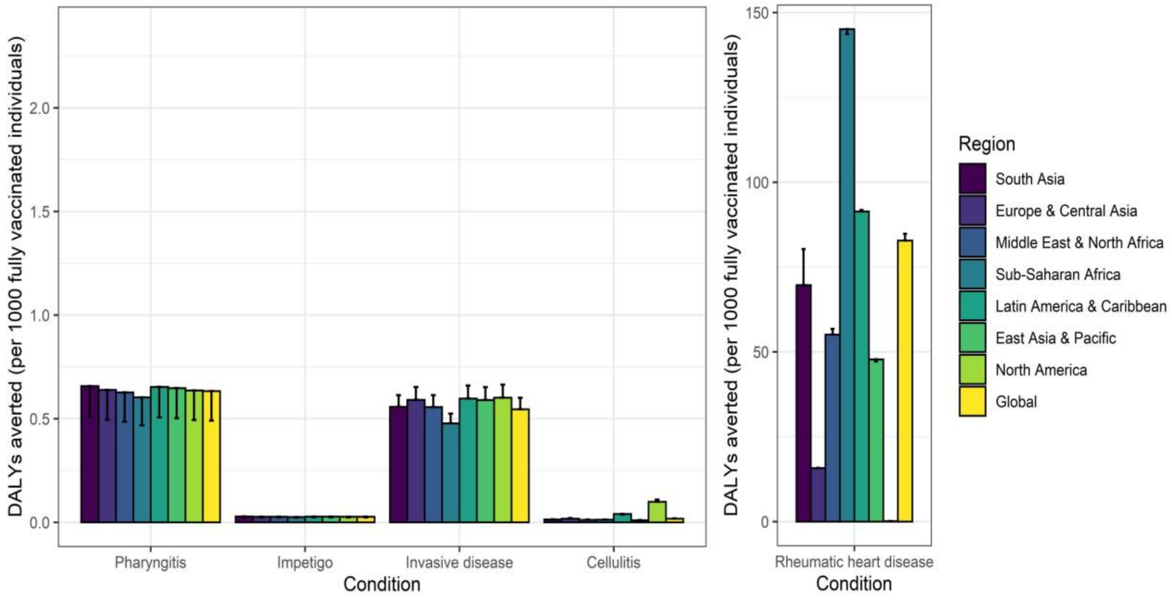

Supplementary Figure 4. Vaccine impact (deaths averted per 1,000 fully vaccinated individuals) at the regional and global levels. The vaccine impact on deaths averted per 1,000 fully vaccinated individuals is stratified at the regional (United Nations regions) and global levels for different scenarios (estimate for scenario 1 and range across the six scenarios), based on the lifetime health impact of vaccination at birth or 5 years of age for 30 vaccinated cohorts on Strep A disease burden that results in death (invasive disease and rheumatic heart disease). The vertical bars show the estimates for scenarios 1, 3, and 5 (which are equal), and the error bars show the estimates for scenarios 2, 4, and 6 (which are equal).

Vaccination at birth

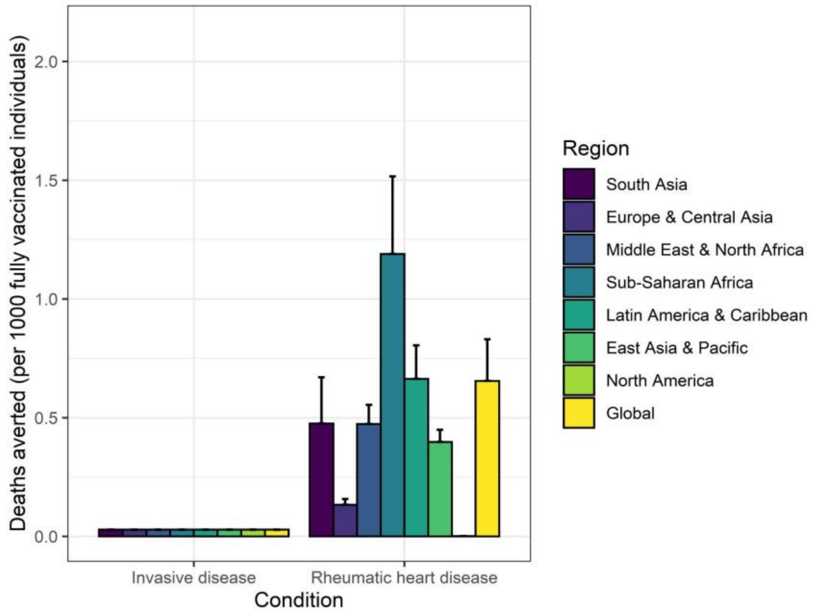

Vaccination at 5 years of age

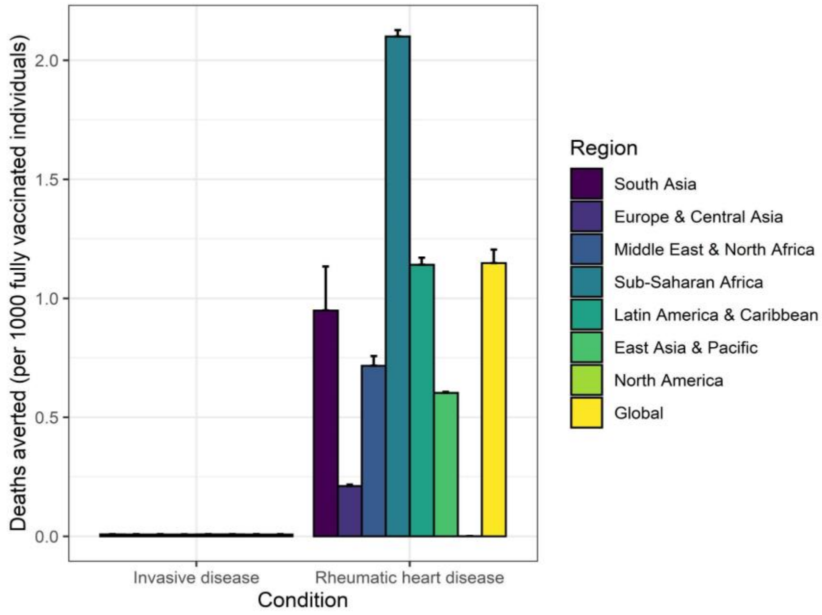

Supplementary Figure 5. Vaccine impact (cases averted per 1,000 fully vaccinated individuals) at the national level. The vaccine impact on cases averted per 1,000 fully vaccinated individuals is shown for 183 countries, based on the lifetime health impact of vaccination at birth or 5 years of age for 30 vaccinated cohorts on Strep A disease burden (pharyngitis, impetigo, invasive disease, cellulitis, and rheumatic heart disease) for scenario 1.

Vaccination at birth

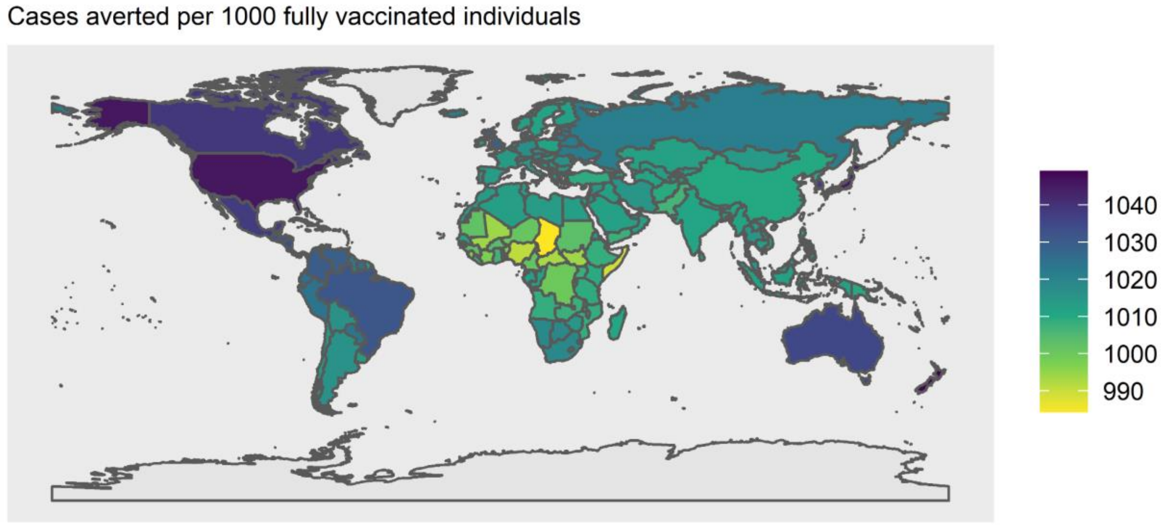

Vaccination at 5 years of age

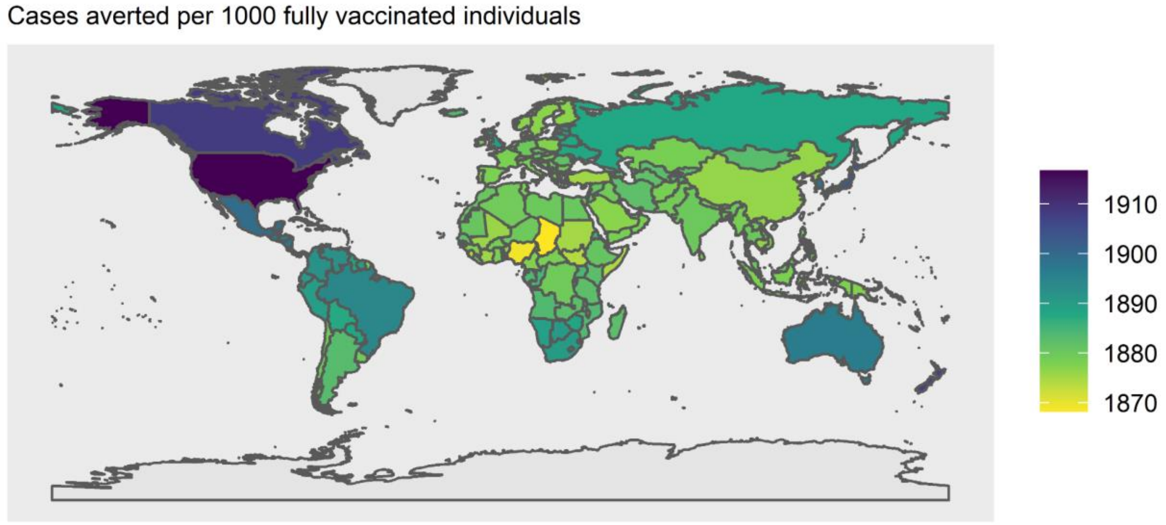

Supplementary Figure 6. Vaccine impact (deaths averted per 1,000 fully vaccinated individuals) at the national level. The vaccine impact on deaths averted per 1,000 fully vaccinated individuals is shown for 183 countries, based on the lifetime health impact of vaccination at birth or 5 years of age for 30 vaccinated cohorts on Strep A disease burden resulting in death(invasive disease and rheumatic heart disease) for scenario 1.

Vaccination at birth

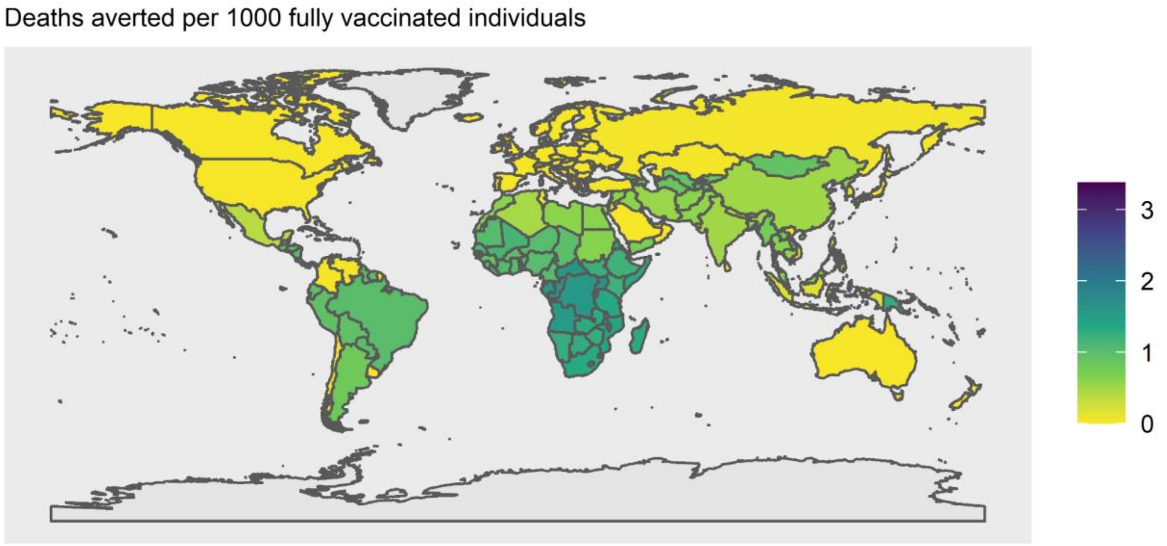

Vaccination at 5 years of age

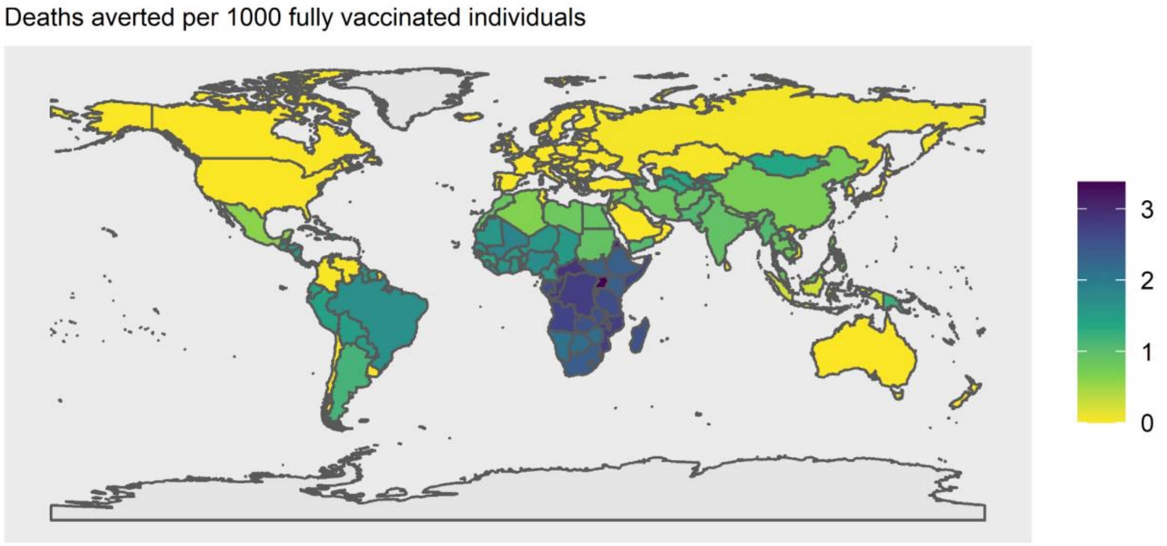

Supplement: Supplementary file 1 — Supplementary Information [file 41541_2023_668_MOESM1_ESM.pdf]
